# Cuproptosis‐Related Genes in Immune Infiltration and Diagnosis in Hepatitis B Virus‐Related Acute Liver Failure

**DOI:** 10.1002/EXP.20240267

**Published:** 2026-03-19

**Authors:** Jingwen Deng, Tao Zhu, Yueyang Wang, Sitong Zhang, Hong Deng, Guilan Quan, Tingting Peng, Chuanbin Wu, Huiqiang Cai, Chao Lu, Xiaopeng Cai

**Affiliations:** ^1^ Department of Medical Oncology Sir Run Run Shaw Hospital School of Medicine Zhejiang University Hangzhou China; ^2^ Department of Pathology Sir Run Run Shaw Hospital School of Medicine Zhejiang University Hangzhou China; ^3^ Research Unit of Intelligence Classification of Tumor Pathology and Precision Therapy Chinese Academy of Medical Sciences and Peking Union Medical College Hangzhou China; ^4^ Department of Pathology Peking Union Medical College Hospital Chinese Academy of Medical Sciences and Peking Union Medical College Beijing China; ^5^ Hepatobiliary and Pancreatic Surgery The Second Affiliated Hospital Zhejiang University School of Medicine Hangzhou China; ^6^ Zhejiang Key Laboratory For Disease Proteomics Department of Pathology School of Medicine Zhejiang University Hangzhou China; ^7^ State Key Laboratory of Bioactive Molecules and Druggability Assessment Guangdong Basic Research Center of Excellence for Natural Bioactive Molecules and Discovery of Innovative Drugs College of Pharmacy Jinan University Guangzhou China; ^8^ Department of Medical Oncology West German Cancer Center University Hospital Essen Essen Germany

**Keywords:** acute liver failure, bioinformatics, cuproptosis, ferroptosis, hepatitis B virus, immune infiltration

## Abstract

Hepatitis B virus (HBV) infection poses a significant challenge to global health, particularly in developing countries such as China, where HBV‐related acute liver failure (HBV‐ALF) is a prominent cause of acute liver failure. This study investigated the effect of cuproptosis, a recently identified form of cell death, on immune infiltration in HBV‐ALF. We mined the gene expression data of HBV‐ALF from the Gene Expression Omnibus database. Through enrichment analysis of differentially expressed genes (DEGs), pathways related to the response to metal/copper ions and the acute inflammatory response were found to be enriched. We subsequently found that HBV‐ALF tissues contained more copper ions and conducted an intersection analysis of DEGs and cuproptosis‐related genes (CRGs), which resulted in the identification of 7 core cuproptosis‐related DEGs (CR‐DEGs) for further investigation with a diagnostic model. Immune infiltration analysis and unsupervised clustering analysis revealed distinct patterns in HBV‐ALF and the possibility of crosstalk between ferroptosis and cuproptosis. Furthermore, we identified 17 transcription factors, 90 miRNAs, and 15 drugs that might interact with the 7 CR‐DEGs. To validate our findings and their clinical significance, we verified the diagnostic value and immune infiltration patterns of the 7 CR‐DEGs in both the testing dataset, cell line, and clinical samples. In conclusion, our findings indicated that these 7 CR‐DEGs demonstrate promising diagnostic value and may represent viable therapeutic targets for individuals with HBV‐ALF.

## Graphical abstract

The role of cuproptosis in HBV‐ALF was explored via bioinformatics and liver biopsy tissues. There are some crosstalks among cuproptosis, immune infiltration, and ferroptosis (The figure was created by Bio Render).

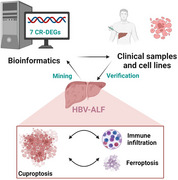

**Abbreviations**
ALF: acute liver failure
BRCA1: BRCA1 DNA repair associated
CDK1: cyclin‐dependent kinase 1
CR‐DEGs: cuproptosis‐related DEGs
CRGs: cuproptosis‐related genes
DEGs: differentially expressed genes
DGIDB: drug‐gene interactions
DLAT: dihydrolipoamide S‐acetyltransferase
GEO: Gene Expression Omnibus
GO: Gene Ontology
GSEA: Gene Set Enrichment Analysis
HBV: hepatitis B virus
HBV‐ALF: HBV‐related ALF
IDH2: isocitrate dehydrogenase 2
KEGG: Kyoto Encyclopedia of Genes and Genomes
LASSO: least absolute shrinkage and selection operation
LT: liver transplantation
MCC: maximal clique centrality
MCM5: minichromosome maintenance protein 5
NDUFS1: NADH dehydrogenase ubiquinone Fe‐S protein 1
PCA: principal component analysis
PDHX: pyruvate dehydrogenase protein X
PPI: protein‐protein interaction
PPRC1: PPARG Related Coactivator 1
ROC: receiver operating characteristic
TFs: transcription factors
TKT: transketolase

## 1 Introduction

Acute liver failure (ALF) is a serious and rare syndrome characterized by sudden damage to hepatocytes, which can lead to the deterioration of liver function, multiple organ failure, and even death [1]. Various factors contribute to ALF, including drugs, viruses, and ischemia. Acetaminophen is the main cause of ALF in developed countries; however, hepatitis B virus (HBV) infection remains the most common cause of ALF in developing countries [2, 3]. There are approximately 257 million chronic HBV‐infected patients worldwide, and more than 0.5% of HBV‐infected patients develop HBV‐related ALF (HBV‐ALF), which leads to 887,000 deaths annually [4, 5]. Effective therapies for ALF are still lacking, and despite antiviral treatment and other supportive measures, emergency liver transplantation (LT) is the only therapeutic method. However, the shortage of donors and dramatic postoperative complications limit the application of LT in HBV‐ALF. Therefore, investigation of the mechanisms and new treatment options for HBV‐ALF seems to be a desirable research topic.

At present, the pathological changes associated with HBV‐ALF have not been elucidated. In recent years, many studies have reported that virus‐specific T‐cell exhaustion contributes to HBV‐ALF, and humoral immunity, including plasma cells and B lymphocytes, also plays a role in its pathogenesis [5, 6, 7]. Moreover, studies have shown that cytokines, such as IL‐1β and IL‐18, activate the NF‐κB pathway, which results in hepatocellular apoptosis in ALF [8, 9]. Oriel et al. reported that the activation and fibrogenesis of hepatic stem/progenitor cells were positively associated with liver necrosis in patients with HBV‐ALF [10]. However, the specific role of the immune system in HBV‐ALF has not been thoroughly studied. Many types of cell death, including ferroptosis, apoptosis and necroptosis, are involved in ALF [11, 12, 13]. The regulation of liver cell death may be a promising strategy for treating HBV‐ALF.

Recently, a novel form of cell death that is distinct from other known types of cell death was identified as cuproptosis [14]. Excess intracellular copper directly targets and binds to lipoylated components of the tricarboxylic acid cycle, which leads to the aggregation of lipoylated proteins and the loss of iron‒sulfur cluster proteins. Proteotoxic stress and cuproptosis subsequently occur [15, 16]. The liver, which is the organ with the highest copper content, predominantly regulates copper homeostasis. The transmembrane Cu‐transporting P‐type ATPase ATP7b transports cytosolic copper for incorporation into ceruloplasmin, which is secreted into the blood [17]. Dysfunction of ATP7b can lead to persistent overload of free copper concentrations, which results in oxidative stress, inflammation, and mitochondrial dysfunction. Fibrosis, cirrhosis, and liver failure in Wilson's disease serve as important models for studying copper homeostasis dysfunctions, which underscores the importance of liver regulation in response to copper [18]. Recent studies have shown that cuproptosis is involved in several liver diseases, especially HBV‐ALF. For example, Liu et al. reported that cuproptosis may contribute to nonalcoholic fatty liver disease and identified 3 characteristic genes via machine learning and external dataset validation [19]. Another study explored the association of cuproptosis with hepatic ischemia‐reperfusion injury (HIRI), which established a promising diagnostic pattern and identified latent therapeutic targets for HIRI treatment [20]. In addition, systematic analyses of the relationships between cuproptosis and other diseases have been conducted in oncology and nononcology areas, such as liver cancer, melanoma, arthritis, and inflammatory bowel disease [21, 22, 23, 24]. Despite these advancements, a more comprehensive comprehension of the role of cuproptosis in HBV‐ALF is necessary, indicating a requirement for further research in this domain.

Recent advancements in the use of machine learning models for analyzing complex biological data have proven to be beneficial in various areas, such as disease diagnosis, drug screening, and basic research [25, 26]. This study conducted a systematic analysis of cuproptosis and immune infiltration in HBV‐ALF. Gene expression data from HBV‐ALF patients were downloaded and subjected to functional enrichment analysis. Our study revealed enrichment of pathways related to the response to metal/copper ions in patients with HBV‐ALF, followed by further investigation into the potential relationship between cuproptosis and HBV‐ALF. The core cuproptosis‐related genes (CRGs) in HBV‐ALF, which exhibited a positive correlation with immune responses, were identified through data mining. Subsequently, these CRGs were examined for the expression levels in another mRNA‐Seq dataset. Cell model of cuproptosis and liver samples specifically collected from HBV‐ALF patients were evaluated to further validate our mining data. Importantly, we also predicted potential transcription factors, miRNAs, and drugs that might interact with these CRGs. These findings have the potential to inform the development of innovative therapeutic strategies for HBV‐ALF by targeting cuproptosis.

## 2 Materials and Methods

### 2.1 Data Acquisition and Processing

Gene expression data of HBV‐ALF and normal samples were mined from the Gene Expression Omnibus database (GEO) (https://www.ncbi.nlm.nih.gov/geo/): GSE38941 (10 normal liver tissues and 17 HBV‐ALF tissues) and GSE96851 (17 normal liver tissues and 17 HBV‐ALF tissues). All datasets were normalized using the “limma” R package [27]. GSE38941 was chosen as a training set, and GSE96851 was selected as a testing set. We identified differentially expressed genes (DEGs) with a |log2‐fold change (FC)| ≥ 1 and adjusted *p* < 0.05. The CRGs included in our study were derived from previous literature [28].

### 2.2 GO and KEGG Enrichment Analyses

Gene Ontology (GO) and Kyoto Encyclopedia of Genes and Genomes (KEGG) pathway analyses were performed using the clusterProfiler R package [29]. A term was considered statistically significant when the adjusted *p* < 0.05.

### 2.3 Identification of Hub CR‐DEGs

Derived from the genome‐wide CRISPR‐Cas9 loss‐of‐function test, 347 CRGs were identified to play a role in cuproptosis based on previous literature [15, 28]. An intersection of DEGs and CRGs was made using the VennDiagram R package. Then, these common genes were uploaded to the STRING database (https://cn.string‐db.org/) to obtain the protein‐protein interaction (PPI) diagram. The interactions with a minimum required score > 0.4 were considered for the construction of the network. We used Cytoscape software v.3.7.1 to identify the top 10 hub genes using the maximal clique centrality (MCC) algorithm. In addition, correlation analysis was also performed on the hub cuproptosis‐related DEGs (CR‐DEGs) using the corrplot package [30]. To determine whether hub CR‐DEGs play a key role in other types of ALF, we further analyzed the expression of genes in public datasets of ischemia/reperfusion injury (GSE14951) and acetaminophen‐induced liver injury (GSE120652).

### 2.4 Construction of a Diagnostic Model of CR‐DEGs

Least absolute shrinkage and selection operation (LASSO) analysis was used to construct a diagnostic model of HBV‐ALF via the glmnet R package [31]. The following parameters were set in the LASSO algorithm: family  =  “binomial”; alpha  =  1; type.measure  =  “deviance”; and nfolds  =  10. The intersection of LASSO and hub CR‐DEGs was used to obtain core CR‐DEGs. Then, receiver operating characteristic (ROC) curves of the single was generated using the R package.

### 2.5 Immune Infiltration Analysis

The infiltration of 22 immune cells in GSE38941 was calculated through the CIBERSORT algorithm (http://cibersort.stanford.edu/) [32]. Then, a principal component analysis was performed to evaluate the differences in immune infiltration between normal and HBV‐ALF tissues. In addition, the correlation between each immune cell was performed using the corrplot package [30]. Finally, we used an estimate algorithm to evaluate the immune scores and stromal purity of HBV‐ALF.

### 2.6 Correlation Analysis Between Core CR‐DEGs and Immune Cell Infiltration

A Spearman analysis of immune cell infiltration and core CR‐DEGs was conducted using the corrplot package [30]. Then, we used a scatter diagram to visually identify the associations.

### 2.7 Subcluster Analysis of Core CR‐DEGs

A hierarchical clustering analysis was performed on the 17 HBV‐ALF samples using the ConsensusClusterPlus package, and the expression profiles of core CR‐DEGs were input as information [33]. Principal component analysis (PCA) was further conducted as described above. Next, DEG analysis of subclusters was performed with the screening criteria of p < 0.05 and |log2 FC| ≥ 1. Then, GO and KEGG analyses and gene set enrichment analysis (GSEA) were conducted to identify the enriched pathways in HBV‐ALF using the clusterProfiler R package [29, 34]. Furtherly, the correlation between core CR‐DEGs and markers of ferroptosis was performed using the corrplot package. Finally, we evaluated the immune infiltration between 2 subclusters using the CIBERSORT algorithm [32].

### 2.8 Construction of TF‐CR‐DEGs, miRNA‐CR‐DEGs, and Drug Interaction Networks

We used the hTFtarget database (http://bioinfo.life.hust.edu.cn/hTFtarget#) to predict transcription factors (TFs) for core CR‐DEGs in liver tissue, which were predicted in at least 2 databases [35]. We used the miRWalk database (http://mirwalk.umm.uni‐heidelberg.de/) to predict miRNAs that were targeted at and bound to core CR‐DEGs based on miRanda, TargetScan, and miRDB. We used the drug‐gene interactions database (DGIDB, www.dgidb.org) to predict drugs that could interact with the core CR‐DEGs. The networks were visualized by Cytoscape software.

### 2.9 Initial Validation of GEO Database, Cell Line, and Clinical Samples

We chose GSE96851 as the testing set and evaluated the expression and diagnostic value of core CR‐DEGs. Then, we conducted immune infiltration analysis in GSE96851 using the CIBERSORT and ESTIMATE algorithms [32].

A stable HBV genome transfected cell line, HepG 2.2.15 cells, was obtained from Dr. Jinjin Qi at the first affiliated hospital of Zhejiang University. HepG 2.2.15 cells were maintained in RPMI 1640 with fetal bovine serum (FBS, GIBCO), and 380 µg/mL G418 at 37 °C (95% humidity and 5% CO2) [36]. To induce cuproptosis in HepG 2.2.15, we added 5, 10, and 20 nM Elesclcomol‐CuCl2 (ES‐Cu, 1:1, MCE, HY‐12040) into cells for 2 h. In the ammonium tetrathiomolybdate (ATTM)‐Elesclomol‐CuCl2 group, HepG 2.2.15 cells were pretreated with 20 µM ammonium tetrathiomolybdate (ATTM, Sigma, 323446) overnight and then treated with 20 nM ES‐Cu for 2 h [37]. Then HepG 2.2.15 cells were cultured with fresh medium for 48h. Cell Counting Kit‐8 (CCK‐8) and microscope were used to evaluate cell viability and cell morphology. Finally, cells were lysed by scraping in RIPA buffer. As in our previous study, we performed a western blot to evaluate the protein expression of 7 core CR‐DEGs. The detailed information on antibodies is shown in Table S1.

To verify the involvement of cuproptosis in HBV‐ALF patients, we first conducted arginine copper staining in HBV‐ALF patients as described in our previous study [3]. We subsequently evaluated the expression of CD45 (a marker of immune infiltration) and 4‐HNE (a marker of ferroptosis described in a previous study) in normal and HBV‐ALF tissues by immunohistochemical staining (IHC) [38]. Finally, we performed IHC to further clarify the changes of CR‐DEGs in HBV‐ALF patients [11, 39]. The clear field of view in the sections was selected, and the immunohistochemical sections were observed and scored by two professional pathologists in a double‐blind method. The frequency score used was immunoreactive score (IRS). We collected the livers of 6 patients with HBV‐ALF and 6 patients with hepatic hemangioma from Sir Run Run Shaw Hospital, School of Medicine, Zhejiang University (ethics committee number: 2023‐926‐01). All patients were informed, and none were involved in the design, conduct, reporting, or dissemination plans of our research.

### 2.10 Statistical Analysis

All data are expressed as the mean ± standard deviation. Data processing and statistical analysis were performed using R software and GraphPad Prism. The Wilcoxon rank‐sum test or Student's t test was used to analyze the differences between 2 groups. The level of statistical significance was set at *p* < 0.05.

## 3 Results

### 3.1 Differences in Functional Enrichment Between Normal and HBV‐ALF Tissues

**FIGURE 1:**
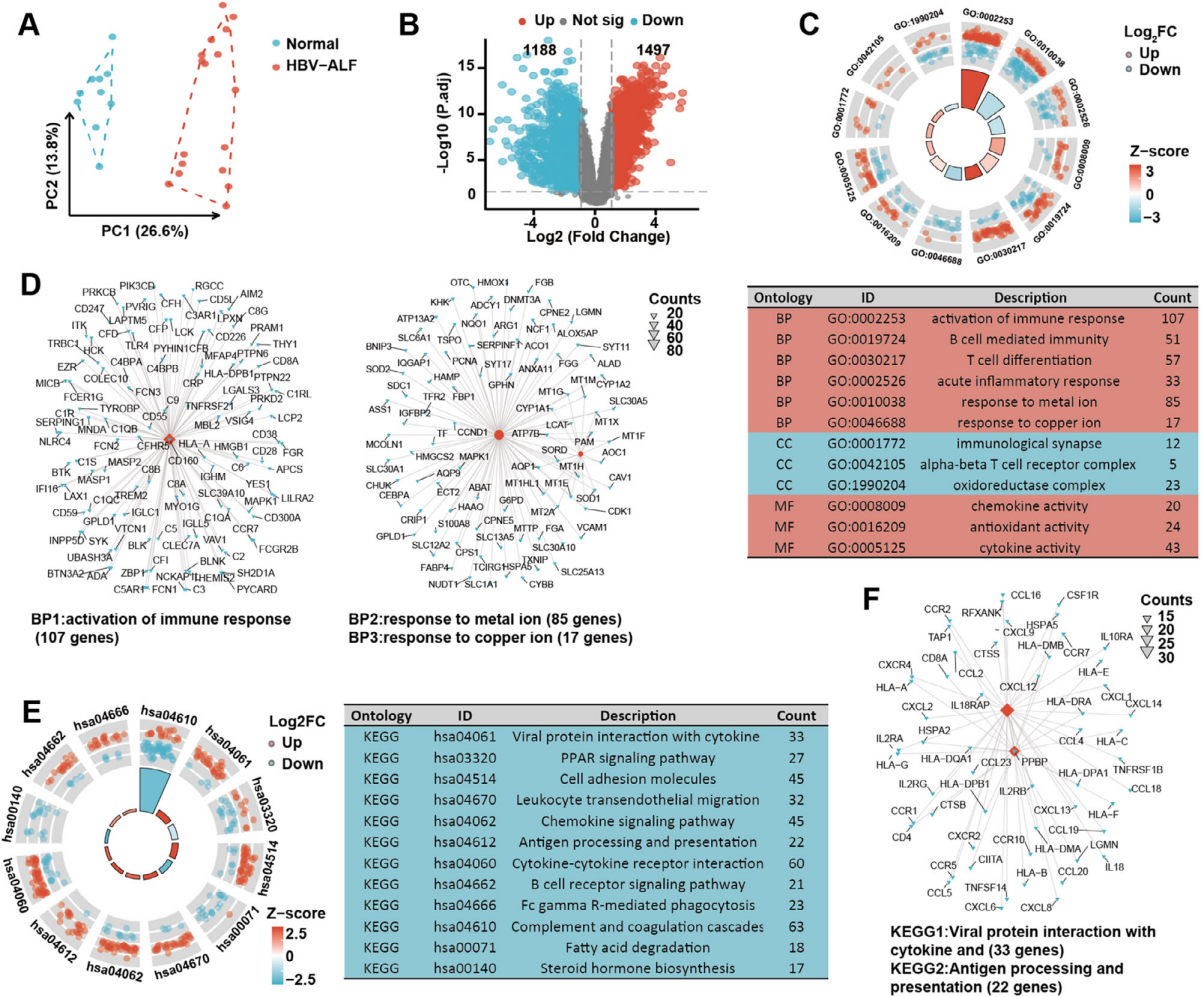
Differences in functional enrichment between normal (n = 10) and HBV‐ALF (n = 17) tissues. (A) Principal component analysis (PCA) based on RNA sequencing data. (B) Volcano plots of DEGs. (C) Gene ontology (GO) analysis of the normal and HBV‐ALF groups based on DEGs. (D) Representative network plot of GO pathways. (E) Kyoto Encyclopedia of Genes and Genomes (KEGG) analysis of the normal and HBV‐ALF groups based on DEGs. (F) Representative network plot of KEGG pathways.

To analyze the RNA sequencing data of normal and HBV‐ALF tissues, we conducted PCA and found that there was an obvious difference between the groups (Figure 1A). Accordingly, analysis of the DEGs revealed 1497 upregulated genes and 1188 downregulated genes. Figure 1B shows the distribution of DEGs. Furthermore, these DEGs were selected for GO and KEGG analyses to explore their biological functions. Figure 1C shows that the pathways of immune response activation, acute inflammatory response, response to metal ions, response to copper ions, and antioxidant activity were enriched. In particular, we constructed two network plots to show the pathways of the immune response and the response to metal/copper ions. Figure 1D shows the 107 genes and 85 genes involved in the two pathways. Moreover, KEGG analysis revealed that several pathways of inflammation induced by viral proteins were enriched (Figure 1E). Figure 1F shows that 33 genes and 22 genes were involved in viral interactions with cytokines and cytokine receptors and antigen processing and presentation, respectively. Overall, these results indicated that inflammation, the immune response, and oxidative stress pathways are enriched in HBV‐ALF. Additionally, abnormalities in copper metabolism may also occur in HBV‐ALF.

### 3.2 Identification of Hub CR‐DEGs

**FIGURE 2:**
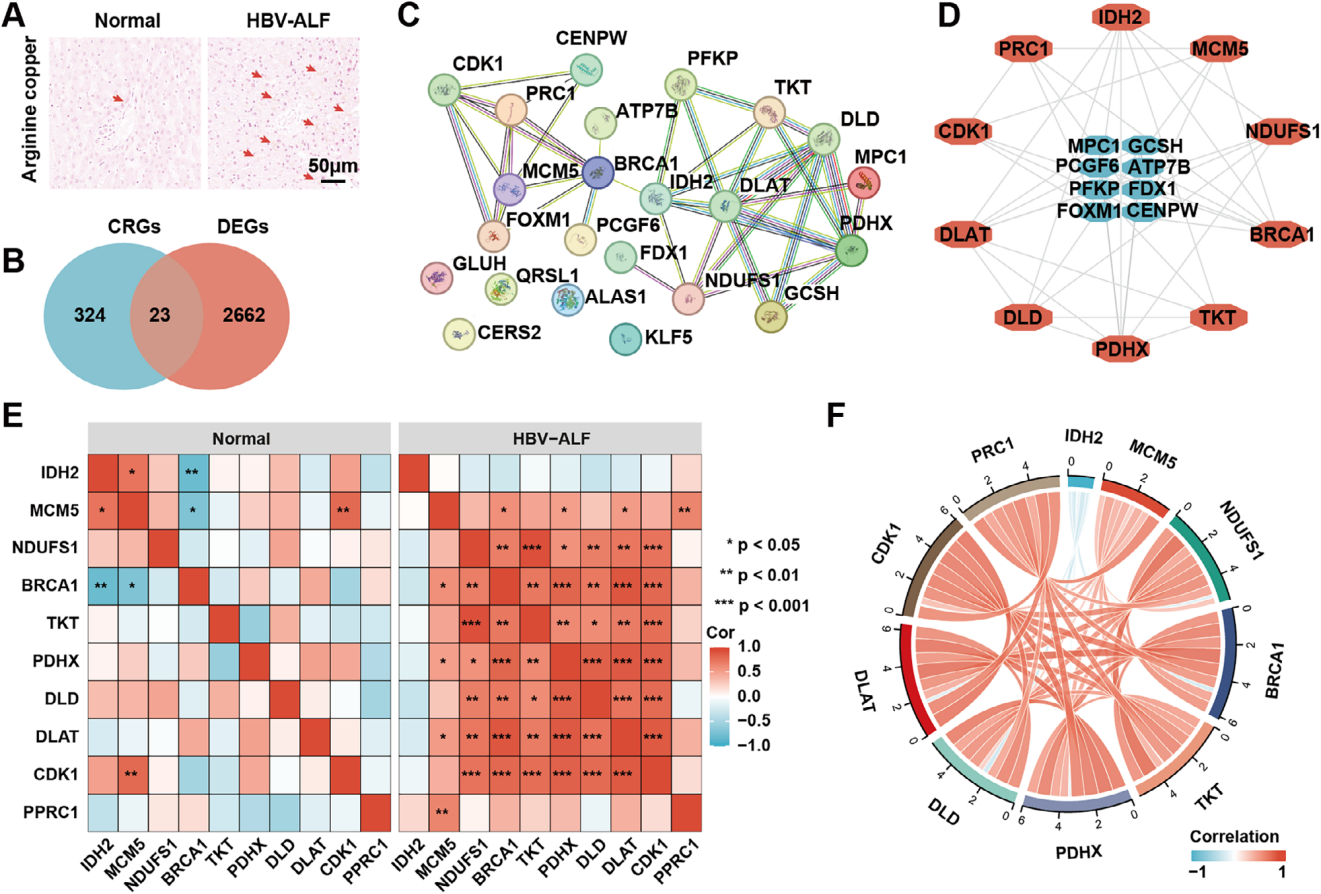
Identification of hub cuproptosis‐related differentially expressed genes (CR‐DEGs) between normal (n = 10) and HBV‐ALF (n = 17) tissues. (A) Arginine copper staining between normal (n = 6) and HBV‐ALF (n = 6) tissues in our cohort. (B) The intersection of 347 cuproptosis‐related genes (CRGs) and DEGs. (C) A protein interaction network of 23 CR‐DEGs. (D) Identification of 10 hub CR‐DEGs via MCC algorithm in Cytoscape software. (E) Correlations among 10 hub CR‐DEGs between normal and HBV‐ALF tissues. (F) Chord plot displaying the relationships of 10 hub CR‐DEGs in HBV‐ALF tissues. ***p < 0.001, **p < 0.01, *p < 0.05.

To verify the involvement of cuproptosis in HBV‐ALF, we used arginine copper staining to evaluate the copper content in HBV‐ALF and normal tissues. As shown in Figure 2A, more copper ions were present in the HBV‐ALF tissues than in the normal tissues, indicating abnormalities in copper metabolism in HBV‐ALF. As described in a previous study, 347 CRGs were identified to play a role in cuproptosis [28]. Here, we examined the intersection of 347 CRGs and 2685 DEGs and identified 23 CR‐DEGs (Figure 2B). To identify the hub CR‐DEGs, we first constructed the PPI network via the STRING website and then identified the 10 hub CR‐DEGs via Cytoscape software (Figure 2C,D). As shown in Figure 2D, DLAT (dihydrolipoamide S‐acetyltransferase), BRCA1 (BRCA1 DNA repair associated), and PDHX (pyruvate dehydrogenase protein X) all interacted with the other 7 molecules, and the other 7 hub CR‐DEGs interacted with at least the other 5 molecules. Next, we evaluated the correlations among the 10 hub CR‐DEGs between normal and HBV‐ALF tissues. As shown in Figure 2E, there were different models of associations between the two groups. Except for *IDH2* (isocitrate dehydrogenase 2), the other 9 genes were positively associated with at least one gene in the HBV‐ALF tissues (Figure 2F). These findings indicated that the progression of cuproptosis may be involved in HBV‐ALF.

### 3.3 The Diagnostic Value of Core CR‐DEGs in HBV‐ALF

**FIGURE 3:**
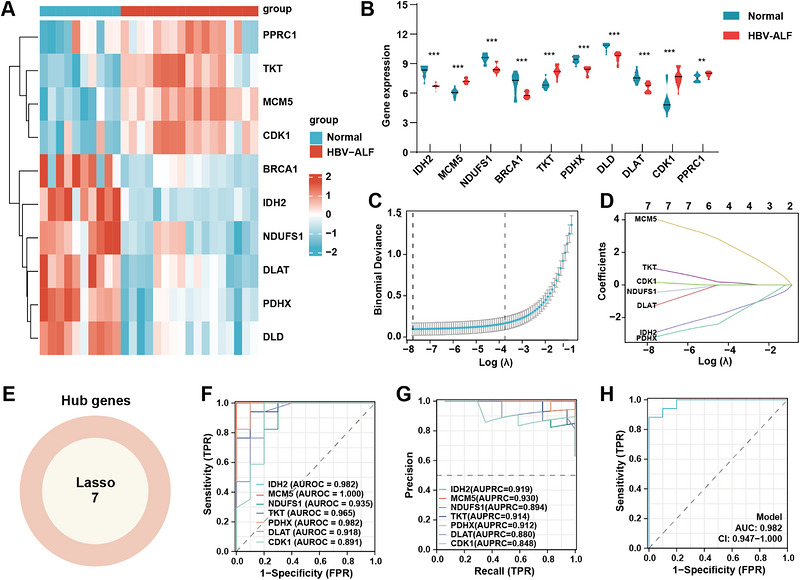
The diagnostic value of core CR‐DEGs in HBV‐ALF. (A) Heatmap of 10 hub CR‐DEGs between normal (n = 10) and HBV‐ALF (n = 17) tissues. (B) Overall expression violin plot of 10 CR‐DEGs. (C) 10 gene expression signatures were selected by the LASSO Cox models. (D) Cross‐validation for tuning parameter selection in the LASSO model. (E) Identification of core CR‐DEGs based on the intersection of 7 featured and 10 hub CR‐DEGs. (F) ROC curve of the 7 core CR‐DEGs in HBV‐ALF diagnosis. (G) Precision and recall curves of the 7 core CR‐DEGs in HBV‐ALF diagnosis. (H) The combined ROC curve of the 7 core CR‐DEGs. ***p < 0.001, **p < 0.01.

We evaluated the expression of 10 hub CR‐DEGs and reported that the levels of *PPRC1* (PPARG‐related coactivator 1)*, TKT* (transketolase)*, MCM5* (minichromosome maintenance protein 5) and *CDK1* (cyclin‐dependent kinase 1) were greater in HBV‐ALF tissues than in normal tissues. The other 6 genes presented opposite expression patterns (Figure 3A,B). To construct the diagnostic model, we performed LASSO analysis to reduce the number of genes. As shown in Figure 3C,D, the optimal lambda value was 0.00043 after tenfold cross‐validation, and the regression coefficients of *IDH2, MCM5, NDUFS1* (NADH dehydrogenase ubiquinone Fe‐S protein 1), *TKT, PDHX, DLAT*, and *CDK1* were ‐2.97, 4.10, −0.47, 1.02, −3.23, −1.32, and 0.18, respectively. Therefore, we identified these 7 hub CR‐DEGs as core CR‐DEGs (Figure 3E). Moreover, we performed a diagnostic value analysis of the 7 core CR‐DEGs with area under the receiver operating characteristic curve (AUROC) using all 27 samples (10 normal liver tissues and 17 HBV‐ALF tissues) under a 10‐fold cross‐validation framework. This analysis revealed that all 7 genes had good diagnostic predictions (Figure 3F). Additionally, we calculated the Area Under the Precision‐Recall Curve (AUPRC) for the random model, which was approximately 0.63 (17/27). Notably, the AUPRC for these 7 genes was significantly higher than 0.63, further confirming the effectiveness of the model (Figure 3G). Finally, we integrated the 7 core CR‐DEGs into a unified predictive score and generated a combined ROC curve, achieving an AUROC value of 0.982, which demonstrates good diagnostic value (Figure 3H). To determine whether the 7 core CR‐DEGs play key roles in other types of ALF, we further analyzed another two datasets with ischemia/reperfusion injury and acetaminophen‐induced liver injury. However, no significant differences were found between the two types of ALF (Table S2).

### 3.4 Immune Infiltration Analysis in HBV‐ALF

**FIGURE 4:**
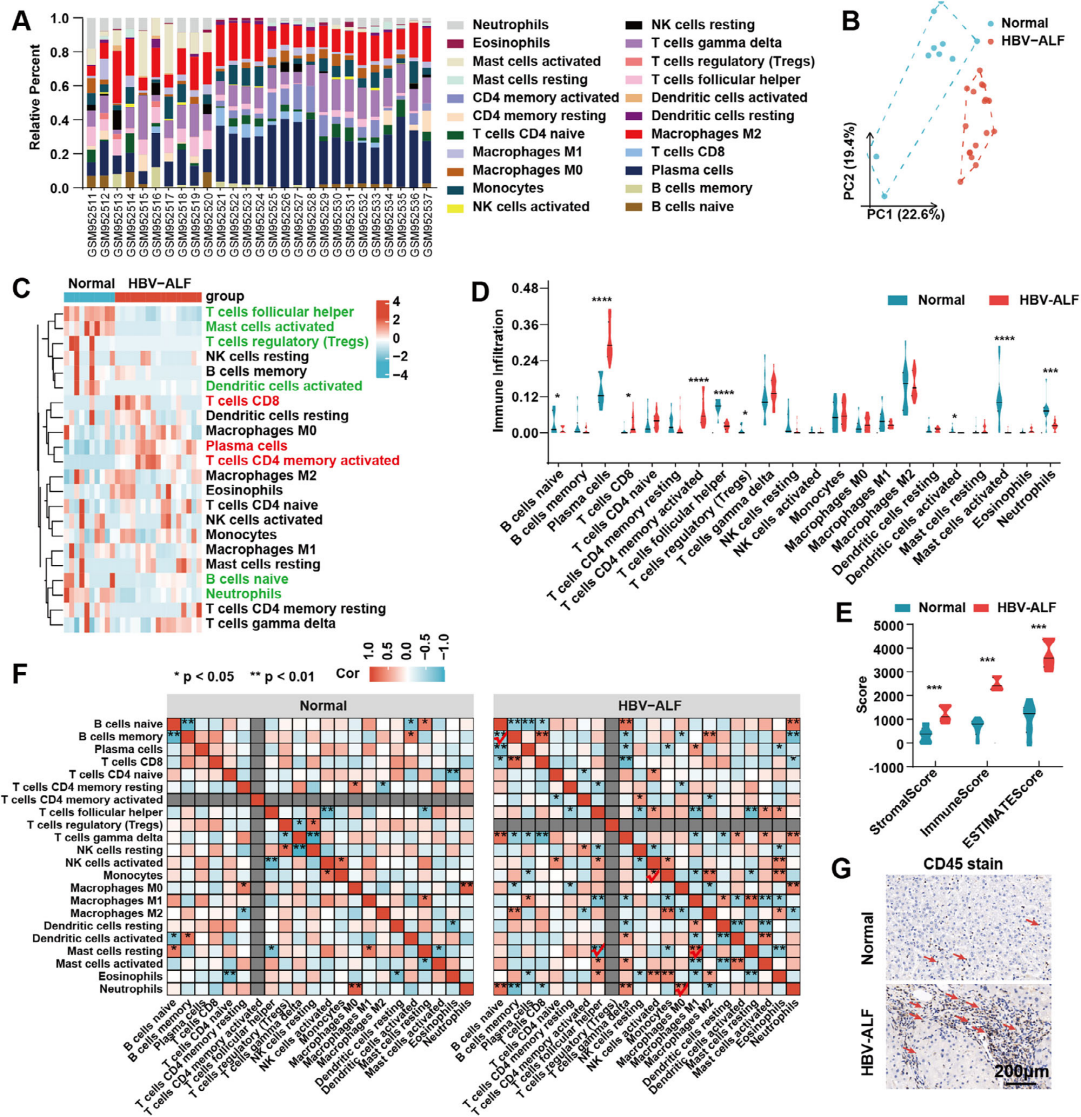
Immune infiltration analysis between normal (n = 10) and HBV‐ALF (n = 17) tissues. (A) Relative levels of infiltrating immune cells in the training dataset. (B) Principal component analysis (PCA) based on immune infiltration. (C) Heatmap of the differences in 22 immune cells between normal and HBV‐ALF tissues. Red indicates an increased abundance and green indicates a decreased abundance in HBV‐ALF tissue. (D) Overall expression violin plot of 22 immune cells via the CIBERSORT algorithm. (E) Immune analysis via the estimate algorithm. (F) Correlation of 22 immune cells between normal and HBV‐ALF tissues. 5 same‐paired associations are marked with red hook. (G) CD45 stain between normal (n = 6) and HBV‐ALF (n = 6) tissues in our cohort. ****p < 0.0001, ***p < 0.001, *p < 0.05.

GO and KEGG analyses revealed that the immune response pathway was enriched in HBV‐ALF. Therefore, we conducted immune infiltration analysis in the GSE38941 dataset by the CIBERSORT method. Figure 4A shows the normalized enrichment score of immune infiltration, which could distinguish the HBV‐ALF group from the normal group (Figure 4B). Figure 4C,D shows the infiltration of 22 immune cells in every sample in the two groups. We found that the levels of plasma cells, CD8 T cells, and activated memory CD4 T cells were increased; however, the levels of naïve B cells, follicular helper T cells, regulatory T cells, activated dendritic cells, activated mast cells, and neutrophils were decreased. Accordingly, the ESTIMATE analysis also revealed that the HBV‐ALF group had a higher immune score (Figure 4E). Further, we subsequently performed an association analysis among the 22 immune cells and identified a different model between normal and HBV‐ALF tissues. We found that HBV‐ALF tissue had only 5 same‐paired associations but 49 different‐paired associations with normal tissue, which indicated the complex interactions involved in HBV‐ALF (Figure 4F). Finally, we evaluated the expression of CD45, a marker of immune infiltration, and found that HBV‐ALF tissues showed a stronger staining than normal tissues did (Figure 4G). Therefore, we concluded that there is a robust immune response in HBV‐ALF.

### 3.5 Correlation Analysis of the 7 Core CR‐DEGs and 22 Immune Cells

**FIGURE 5:**
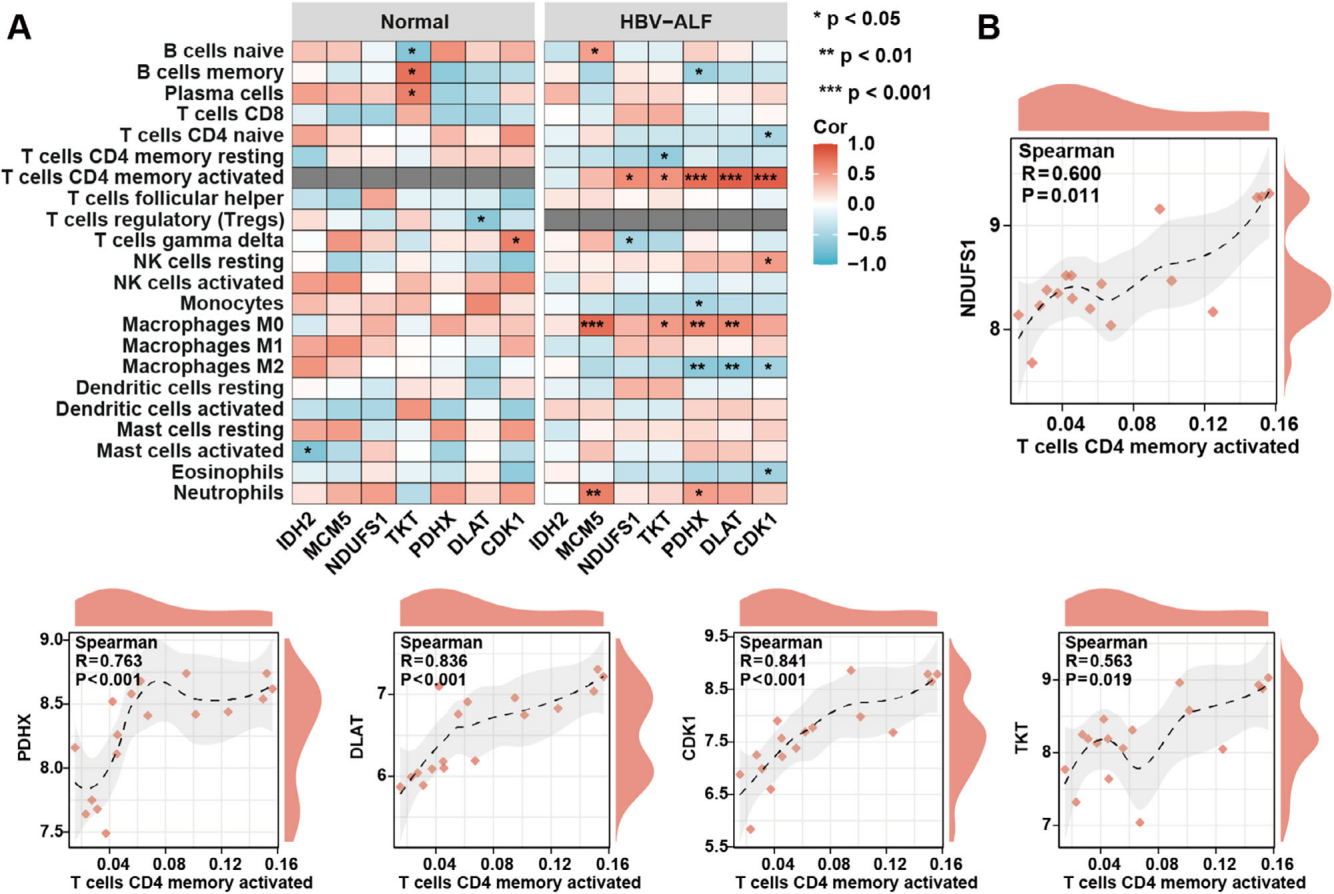
Correlation analysis of 7 core CR‐DEGs and 22 immune cells between normal (n = 10) and HBV‐ALF (n = 17) tissues. (A) Correlation heatmap of the 7 core CR‐DEGs and immune cells. (B) Correlation analysis of NDUFS1, TKT, PDHX, DLAT, CDK1, and activated memory CD4 T cells. ***p < 0.001, **p < 0.01, *p < 0.05.

We subsequently analyzed the correlations between the 7 core CR‐DEGs and 22 immune cell types. As shown in Figure 5A, all 7 genes had different relationships with immune cells. In particular, B‐cell memory cells, CD4 naïve cells, CD4 memory resting cells, M2 macrophages, and eosinophils were negatively associated with the 7 core CR‐DEGs in HBV‐ALF. However, the other associations were positive. Furthermore, we evaluated the consistency between immune cell changes and the associations of the 7 core CR‐DEGs in HBV‐ALF. As shown in Figures 4E and 5A, activated memory CD4 T cells were the only immune cells that were more abundant in HBV‐ALF and were positively associated with *NDUFS1, TKT, PDHX, DLAT*, and *CDK1*. Therefore, we performed Spearman correlation analysis between CD4 memory‐activated T cells and 5 core CR‐DEGs. As shown in Figure 5B, the association coefficients of *NDUFS1, TKT*, and *PDHX* were 0.6, 0.563, and 0.763, respectively, which indicated moderate intensity. The association coefficients of *DLAT* and *CDK1* were 0.836 and 0.841, respectively, which indicated strong intensity. Yao et al. reported that the levels of CD4 lymphocytes from healthy individuals markedly increased when stimulated with plasma containing high HBV copy numbers, which indicated the severity of liver function impairment [40]. Therefore, we hypothesized that activated memory CD4 T cells may be involved in cuproptosis.

### 3.6 HBV‐ALF Unsupervised Clustering Identification and Analysis

**FIGURE 6:**
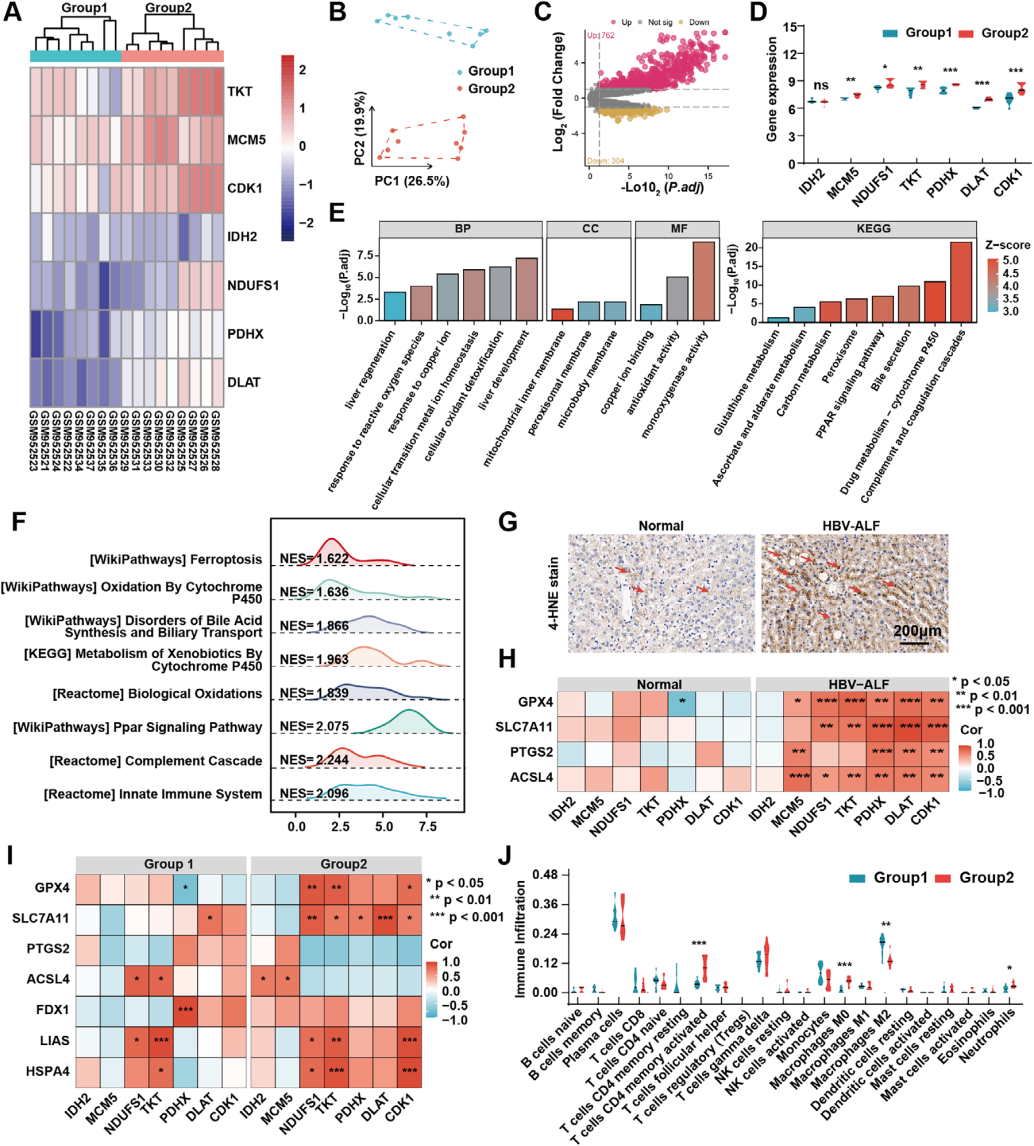
HBV‐ALF unsupervised clustering identification and analysis. (A) Expression heatmap of the 7 core CR‐DEGs in 2 groups (sample sizes: group 1 = 8, group 2 = 9). (B) Principal component analysis (PCA) in 2 groups. (C) Volcano plots of DEGs in 2 groups. (D) Overall expression violin plot of the 7 core CR‐DEGs in 2 groups. (E) GO and KEGG analyses based on DEGs. (F) GSEA analysis based on DEGs. (G) 4‐HNE staining between normal (n = 6) and HBV‐ALF (n = 6) tissues in our cohort. (H) Correlation analysis between the identified markers of ferroptosis (GPX4, SLC7A11, PTGS2, and ACSL4) and the 7 core CR‐DEGs. (I) Correlation analysis between the identified markers of ferroptosis/cuproptosis and the 7 core CR‐DEGs. (J) Overall expression violin plot of 22 immune cells in 2 groups. ***p < 0.001, **p < 0.01, *p < 0.05.

To evaluate whether the 7 core CR‐DEGs can discriminate HBV‐ALF, we used a hierarchical clustering algorithm to discern the 17 HBV‐ALF samples by the expression of the 7 core CR‐DEGs. As shown in Figure 6A, we divided the HBV‐ALF samples into two groups: group 1 (N = 8) and group 2 (N = 9). The PCA plot revealed that the two groups were significantly different (Figure 6B). We identified 1066 DEGs (|log2 FC| ≥ 1 and *p* < 0.05), including 762 upregulated genes and 304 downregulated genes (Figure 6C). The violin plot revealed that there was no significant difference in the *IDH2* expression levels between the two groups; however, the expression levels of the other 6 core CR‐DEGs were greater in group 2 than in group 1 (Figure 6D). GO and KEGG analyses and GSEA were subsequently performed to determine the characteristics of the two groups. GO and KEGG analyses revealed that the pathways of cellular oxidant detoxification, cellular transition metal ion homeostasis, response to copper ions, and glutathione metabolism were enriched (Figure 6E). Similarly, GSEA revealed that the innate immune system, biological oxidation, and ferroptosis pathways were enriched (Figure 6F). Furthermore, we performed staining for 4‐HNE, a marker of ferroptosis, and found that the staining of HBV‐ALF tissues was greater (Figure 6G). Moreover, correlation analysis between the identified markers of ferroptosis (GPX4, SLC7A11, PTGS2, and ACSL4) and the 7 core CR‐DEGs revealed that HBV‐ALF tissues showed a stronger correlation, indicating the involvement of ferroptosis in HBV‐ALF (Figure 6H). In addition, we found that group 2 had a stronger correlation with markers of 2 modes of cell death (identified markers of cuproptosis included FDX1, LIAS, and HSPA4; Figure 6I). Finally, we evaluated the immune infiltration levels in the two groups and found that group 2 had more activated memory CD4 T cells and neutrophils and fewer M2 macrophages than did group 1 (Figure 6G). Studies have shown that M2 macrophages are anti‐inflammatory cells and that neutrophils are harmful in liver diseases [41]. Overall, we concluded that group 2 had a more robust immune response and increased oxidative stress.

### 3.7 The Network of TF‐CR‐DEGs, miRNA‐CR‐DEGs, and Drug Interactions

**FIGURE 7:**
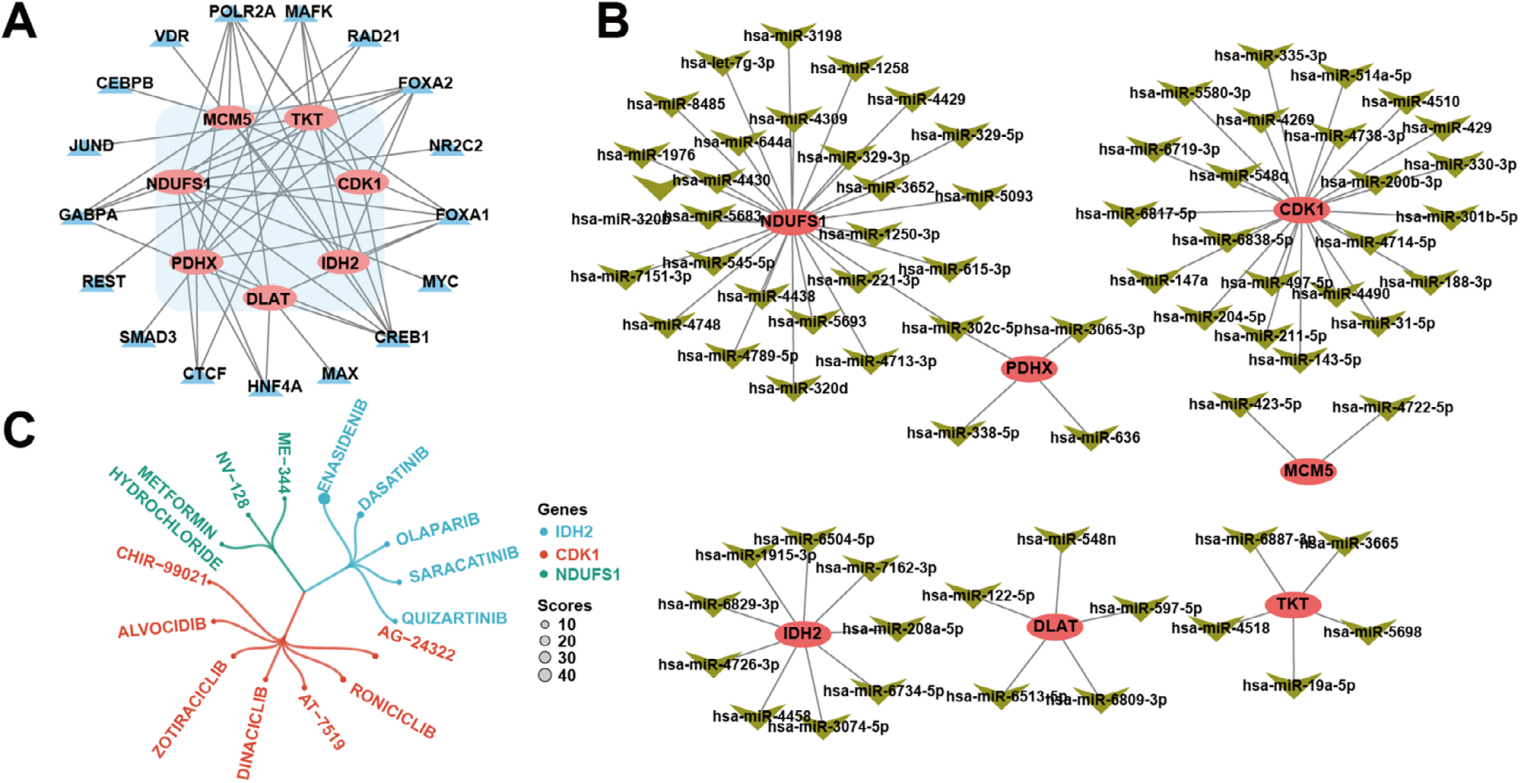
The network of transcription factor (TF)‐CR‐DEGs, miRNA‐CR‐DEGs, and drug interactions. (A) TF prediction of 7 core CR‐DEGs. (B) miRNA prediction. (C) Prediction of drug‒gene interactions.

To further explore the molecular interaction mechanisms between TFs/miRNAs and the 7 core CR‐DEGs, we predicted the potential TFs and miRNAs via the hTFtarget and hTFtarget database databases and then created TF‒mRNA network and miRNA‒mRNA network plots (Figure 7A,B). As the plots show, the TFs and miRNAs of 7 genes had both exclusive TFs/miRNAs and shared phenomena. Thus, these 7 core CR‐DEGs might be involved in the same regulatory process and have similar biological functions. Moreover, these multiple binding sites for TFs/miRNAs could guide further studies on their mechanism. Next, we performed drug‒gene interaction analysis and found that 15 drugs could interact with IDH2, CDK1, and NDUFS1. The interaction between IDH2 and enasidenib had the highest score (Figure 7C). These results revealed the possibility of designing drugs that target the core CR‐DEGs.

### 3.8 Initial Validation of the 7 Core CR‐DEGs in HBV‐ALF

**FIGURE 8:**
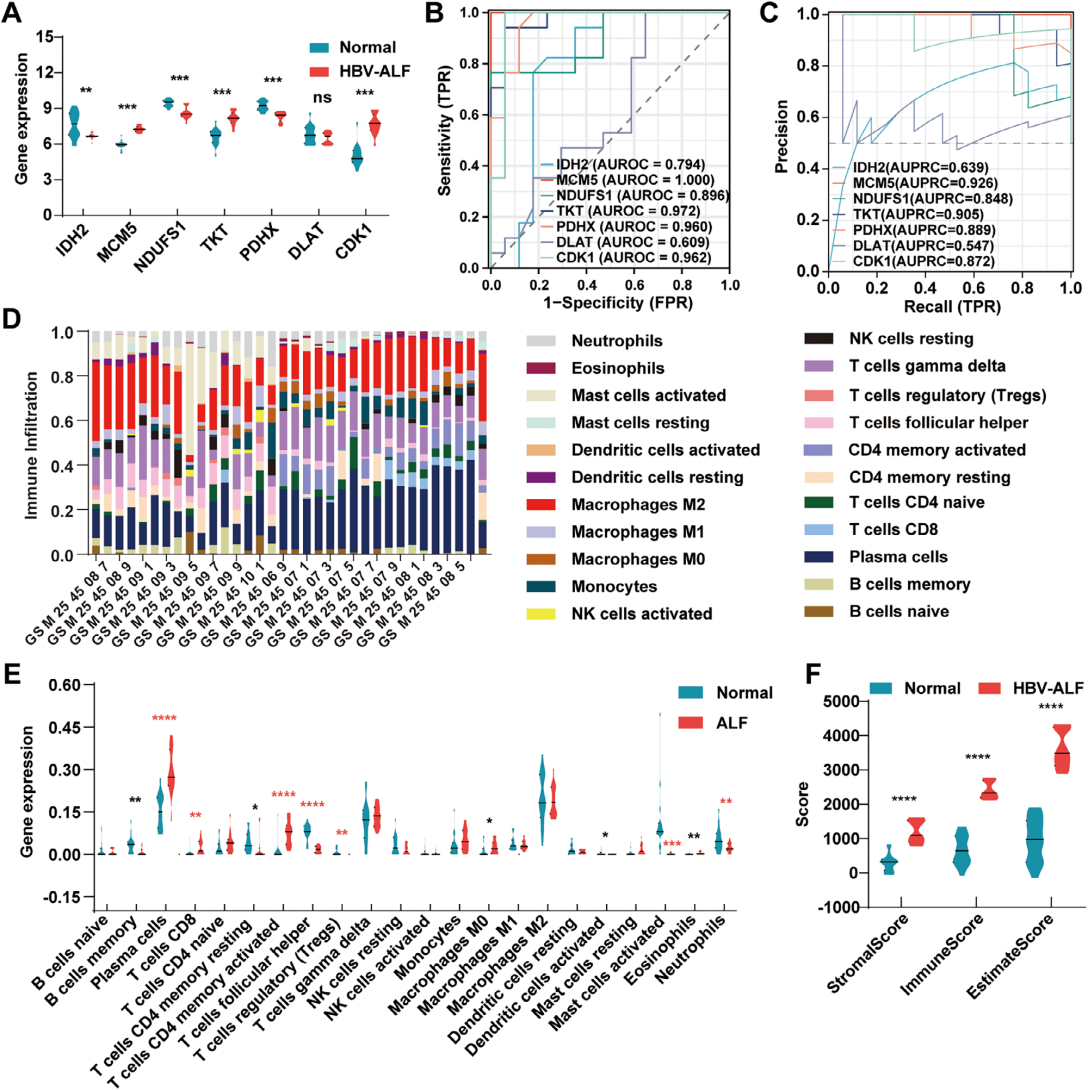
Initial validation of the 7 core CR‐DEGs in the testing dataset between normal (n = 17) and HBV‐ALF (n = 17) tissues. (A) Overall expression violin plot of the 7 core CR‐DEGs. (B) ROC curve of the 7 core CR‐DEGs. (C) Precision and recall curves of the 7 core CR‐DEGs. (D) Relative levels of the infiltrating immune cells. (E) Overall expression violin plot of 22 immune cells via CIBERSORT algorithm. (F) Immune analysis via the estimate algorithm. ****p < 0.0001, ***p < 0.001, **p < 0.01, *p < 0.05.

First, we chose the GSE96851 dataset to validate the above findings. The levels of *IDH2, MCM5, NDUFS1, TKT, PDHX*, and *CDK1* showed the same changes in HBV‐ALF tissues as those in the GSE38941 dataset did, but the changes in the levels of *DLAT* were not the same (Figure 8A). The AUROC of the 7 core CR‐DEGs also demonstrated excellent diagnostic performance (Figure 8B). The AUPRC of the random model was approximately 0.5 (17/34), while the AUPRC of the 7 core CR‐DEGs exceeded 0.5, further highlighting the effectiveness of the model (Figure 8C). Furthermore, we evaluated immune infiltration in the GSE96851 dataset. Figure 8D shows the normalized enrichment score of immune infiltration. We found that the 7 immune cells demonstrated the same changes, including changes in plasma cells, CD8 T cells, activated memory CD4 T cells, follicular helper T cells, Tregs, activated mast cells, and neutrophils (Figure 8E). However, the filtration patterns of naïve B cells and memory B cells were slightly different. Similarly, the ESTIMATE analysis of the GSE96851 dataset also revealed an increase in the immune score in HBV‐ALF tissues.

**FIGURE 9:**
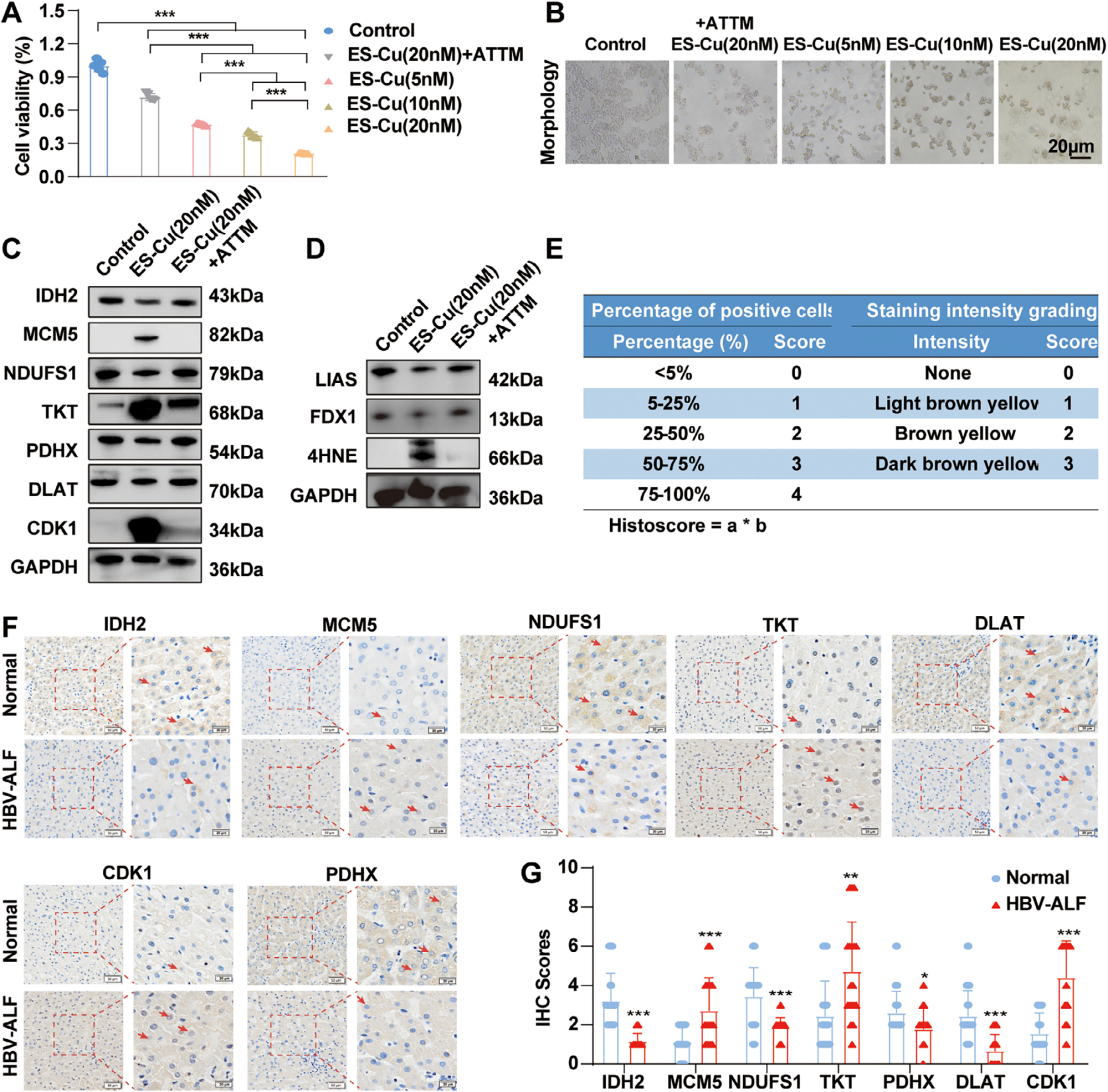
Validation of the 7 core CR‐DEGs in cell line and HBV‐ALF samples. (A) Cell viability (n = 5) and (B) cell morphology (n = 3) were detected after the mono or combined treatment of ATTM and Elesclcomol‐CuCl_2_ (ES‐Cu). (C) 7 CR‐DEGs were evaluated by western blot after co‐culture (n = 3). (D) The western blot of LIAS, FDX1 and 4HNE (n = 3). (E) The detailed scoring rule for IHC. (F) Representative images and (G) IHC scores of the 7 core CR‐DEGs between normal (n = 6) and HBV‐ALF (n = 6) tissues in our cohort. ***p < 0.001, **p < 0.01, *p < 0.05.

Furthermore, we conducted a cuproptosis HepG 2.2.15 cell model to simulate HBV‐ALF‐associated cell death. After co‐culture with the cuproptosis inducer ES‐Cu, we observed that HepG 2.2.15 cell proliferation was inhibited, and the cells were significantly damaged and lysed (Figure 9A,B). Notably, ATTM helped to recover the injured cells from damage. We also examined the protein expression of the 7 core CR‐DEGs following cuproptosis. Consistent with former findings, MCM5, TKT, and CDK1 exhibited higher expression, while the others showed lower expression in the ES‐Cu treatment group (Figure 9C). Moreover, definite known proteins that inhibit cuproptosis, such as LIAS and FDX1, decreased in the ES‐Cu treatment group. We also found that 4HNE levels increased, indicating a potential crosstalk between ferroptosis and cuproptosis (Figure 9D).

We subsequently collected liver samples from 6 HBV‐ALF patients and used liver samples from 6 hepatic hemangioma patients as controls in a clinical setting to evaluate the expression levels of these 7 core CR‐DEGs by IHC (the detailed IRS is shown in Supplementary File 1). Figure 9E shows the detailed scoring rule for IHC. Our findings revealed that MCM5, TKT, and CDK1 were highly expressed in HBV‐ALF patients, whereas IDH2, NDUFS1, PDHX, and DLAT were downregulated (Figure 9F,G). The consistent expression patterns of these 7 CR‐DEGs between the clinical samples and the GSE38941/GSE96851 datasets suggest that they possess significant diagnostic value and contribute to a unique immune response model specific to HBV‐ALF.

## 4 Discussion

HBV‐ALF is the main cause of ALF, which can lead to a sharp decline in liver function and increased mortality in severe cases [4]. Therefore, precise diagnosis and targeted treatment may reverse poor outcomes. To date, the pathogenesis of HBV‐ALF is still not fully understood, but the immune system was found to play a crucial role in HBV‐ALF patients with chronic cirrhosis [42]. Additionally, there is increasing evidence of the integral roles of cuproptosis, a novel form of programmed cell death, in immune responses in various diseases [22, 43, 44, 45]. In particular, cuproptosis influences the tumor microenvironment, and cuproptosis regulators are believed to be potential therapeutic strategies beyond traditional therapy in cancers [43, 46, 47]. Therefore, cuproptosis may play a role in immune responses during liver failure. In this study, bioinformatics was used to determine the diagnostic value of CR‐DEGs, the different infiltration patterns of immune cells, and their biological functions in HBV‐ALF.

First, it was observed that HBV‐ALF tissues contained more copper ions than normal tissues, indicating abnormalities in copper metabolism. Therefore, we further clarified the role of cuproptosis in HBV‐ALF via bioinformatics. We found that there were different gene expression patterns between normal and HBV‐ALF tissues and identified a total of 2685 DEGs, of which 1188 were downregulated and 1497 were upregulated. The GO and KEGG enrichment analyses revealed enrichment of immune response activation, viral interactions with cytokines and cytokine receptors, antigen processing and presentation, response to metal/copper ions, and antioxidant activity. Bioinformatic analysis provides a way to understand the mechanisms underlying cuproptosis and disease [21, 22, 23, 24, 43, 44]. We subsequently obtained a total of 23 CR‐DEGs through the intersection of the DEGs with 347 CRGs [28]. To determine the most important hub CR‐DEGs, we constructed a PPI interaction network and used the MCC algorithm. Finally, *IDH2, MCM5, NDUFS1, BRCA1, TKT, PDHX, DLD, DLAT, CDK1*, and *PRC1* were identified as 10 hub CR‐DEGs. These hub genes were all positively associated with each other in HBV‐ALF, except for *IDH2*. This correlation might indicate a regulatory relationship among the CR‐DEGs. Moreover, we performed LASSO analysis to construct a diagnostic model based on the 10 hub genes, and 7 genes were verified to have excellent diagnostic value for HBV‐ALF.

The human hepatocarcinoma cell line HepG2.2.15, which contains a stably integrated 1.3‐copy HBV genome, was commonly used to simulate an HBV‐infected cell model. We conducted cuproptosis HepG2.2.15 cell model, and observed that ES‐Cu treatment induced the expression of MCM5, TKT, and CDK1 in HepG2.2.15 cells, while IDH2, NDUFS1, PDHX, and DLAT showed decreased expression in the ES‐Cu treatment group. Meanwhile, key protein involved in copper regulation, LIAS and FDX1, exhibited decreased expression in the ES‐Cu treatment group. However, ATTM was able to reverse these changes. These preliminary findings suggest the excellent diagnostic values of 7 core CR‐DEGs in HBV‐ALF‐associated cuproptosis.

Next, we reported that *MCM5, TKT*, and *CDK1* levels were higher in HBV‐ALF tissues than in normal tissues. *MCM5* acts as a critical cell cycle regulator and is a key component of the DNA replication licensing system [48]. Several studies have shown that MCM5 is involved in liver development during liver organogenesis and hepatocyte regeneration after partial hepatectomy [49, 50]. *TKT* is a key enzyme in the nonoxidative phase of the pentose phosphate pathway that has been shown to promote the development of hepatocellular carcinoma (HCC) in metabolic and nonmetabolic manners [51, 52]. *CDK1* is an important cell cycle‐regulating protein that is the only CDK that can independently promote the cell cycle [53]. Studies have shown that CDK1 inhibits hepatocyte apoptosis in fulminant liver injury and promotes cell proliferation in HCC [54, 55, 56]. Miguel et al. reported that oxindolimine‐copper and zinc complexes induce tumor cell apoptosis via the inhibition of the kinase CDK1/cyclin B protein, indicating that CDK1 is involved in cuproptosis [57]. In summary, these 3 core CR‐DEGs were found to be associated with hepatocyte growth. Hepatocyte growth and regeneration increased after cuproptosis in HBV‐ALF.

Furthermore, we found that *IDH2, NDUFS1, PDHX*, and *DLAT* levels were decreased in HBV‐ALF tissues. *IDH2* is a key generator of NADPH in mitochondria and is also a major redox regulatory enzyme that prevents oxidative stress [58]. Studies have shown that IDH2 deficiency exacerbates liver damage in nonalcoholic fatty liver disease, acetaminophen‐induced liver injury, and liver ischemia‒reperfusion injury [59, 60, 61]. *NDUFS1*, which is the core subunit of mitochondrial complex I, reverses the formation of reactive oxygen species [62]. NDUFS1 knockdown in the liver and neurons increases ROS and impairs mitochondrial respiration [63, 64]. *PDHX*, which is located in the mitochondrial matrix, catalyzes the conversion of pyruvate to acetyl coenzyme A. Andy et al. reported that PDHX was decreased in cachexic livers, indicating that lactate metabolism and transport changed [65]. Another study revealed that cuproptosis promoted esophageal carcinoma related to heat stress, where PDHX played a role in the malignant characterization of tumor cells [66]. *DLAT* is a part of the pyruvate dehydrogenase complex that is involved in glucose metabolism and the TCA cycle [43]. Studies have shown that DLAT is highly expressed in liver cancer tissues and is positively associated with clinical stage/grade and poor prognosis [67, 68]. DLAT knockdown effectively inhibits cuproptosis [67]. However, Fang et al. reported that paeoniflorin decreased DLAT expression and serum copper, thereby inhibiting cuproptosis in acute myocardial infarction [69]. The opposite expression profiles of DLAT in cancer and inflammation hinted us to further investigate the role of DLAT in HBV‐ALF.

Immune cells, as executors of the immune system, play a key role in virus‒host interactions in HBV‐ALF [70]. Several studies have reported that complement produced by plasma cells and IgG/IgM produced by B lymphocytes accumulate in necrotic areas, which indicates that humoral immunity contributes largely to the pathogenesis of HBV‐ALF [7, 71]. In addition, the immune response mediated by T lymphocytes has been found to be important in liver damage induced by HBV [72]. In addition to the abovementioned adaptive immunity, innate immunity, including cytokines and chemokines, also activates the pathogenesis of ALF [8, 9]. Using bioinformatic mining, Chen et al. reported strong immune cell infiltration in HBV‐ALF, and the levels of 10 of 11 types of immune cells, including T cells, CD8 T cells, cytotoxic cells, NK cells, B cells, monocytes, macrophages, DCs, endothelial cells, and fibroblasts but not neutrophils, were increased [5]. However, Chen's classification of immune cells did not provide more specific details. We subsequently conducted immune infiltration analysis to reveal the immune response in HBV‐ALF. Here, we used the CIBERSORT algorithm to obtain the infiltration of 22 immune cell types. We found that the proportions of immune cells, including plasma cells, CD8 T cells, and CD4 memory T cells, were significantly increased in HBV‐ALF, which indicated that both cellular and humoral immune cells were significantly activated. In contrast, the levels of naïve B cells, follicular helper T cells, regulatory T cells, activated dendritic cells, activated mast cells, and neutrophils were decreased. Neutrophils die quickly if they respond to infection and are short‐lived [73]. Here, we speculated that a massive number of neutrophils die, and thus, the neutrophil level decreases after they carry out their activities, which would induce a more robust inflammatory response. However, the mechanisms and roles of other changed immune cells in HBV‐ALF are not fully understood, and further study is needed. Finally, we verified via CD45 staining that immune infiltration was greater in HBV‐ALF tissues.

In this study, memory B cells, naïve CD4 T cells, resting memory CD4 T cells, M2 macrophages, and eosinophils were negatively associated with core CR‐DEGs in HBV‐ALF. However, naïve B cells, activated memory CD4 T cells, resting NK cells, M0 macrophages, and neutrophils were positively associated with the core CR‐DEGs. Given the consistency of immune infiltration changes and the correlation between CR‐DEGs and immune cells, we found that activated memory CD4 T cells were positively associated with *NDUFS1, TKT, PDHX, DLAT*, and *CDK1*. Recent studies have shed light on the roles of the 7 core CR‐DEGs in influencing immune cell activity. For instance, MCM5 and CDK1 have been observed as adverse prognostic factors in lung adenocarcinoma and HBV‐related hepatocellular carcinoma, respectively. These two molecules are believed to be involved in tumor progression by affecting the tumor immune microenvironment [74, 75]. Wu et al. reported that low *NDUFS1* expression was correlated with poor infiltration of CD4 T cells, indicating unfavorable survival in patients with kidney renal clear cell carcinoma [76]. Another study revealed that genomic alterations in IDH2 resulted in significant changes in immune biomarkers in intrahepatic cholangiocarcinoma [77]. Overall, these results suggest that CR‐DEGs might directly affect immune infiltration in HBV‐ALF, and the potential mechanisms involved need to be validated in future research.

We used unsupervised cluster analysis to identify two distinct cuproptosis‐related clusters in HBV‐ALF. In the subcluster function analyses, we found that the pathways of cellular oxidant detoxification, response to copper ions, the innate immune system, biological oxidation, and ferroptosis were enriched. To date, there have been limited studies directly verifying ferroptosis in HBV‐ALF. However, Shi et al. reported that selenium regulates apoptosis and ferroptosis in different stages of HCC, which may explain its anti‐HBV and anti‐HCC properties. In our study, we selected 4‐HNE, a product of oxidative stress and ferroptosis, as an indicator of ferroptosis and found stronger 4‐HNE staining in HBV‐ALF tissues. Additionally, the correlation analysis between the ferroptosis markers and the 7 core CR‐DEGs revealed a strong effect. Consistent with other studies that have identified crosstalk between different cell death programs in ALF, we also observed a strong correlation between ferroptosis and cuproptosis. Furthermore, we found that the correlation between the identified markers of ferroptosis/cuproptosis and the 7 core CR‐DEGs was stronger in group 2. We also found higher 4‐HNE expression in the cuproptosis HepG2.2.15 cell model. However, the precise relationship between ferroptosis and cuproptosis needs further clarification through the administration of antagonists or agonists in HBV‐ALF animal models. Finally, we found that group 2 had more activated memory CD4 T cells and neutrophils and fewer M2 macrophages, which shows that core CR‐DEGs influence immune infiltration in HBV‐ALF. In summary, our results indicated that the 7 core CR‐DEGs can delineate the characteristics of HBV‐ALF subgroups.

TFs and miRNAs are regulators of biological processes that regulate the expression of multiple targeted genes. On the basis of online prediction websites, researchers can obtain potential TFs and miRNAs for targeted genes, and these research strategies have been widely used in various studies [35, 78]. Here, we identified 17 TFs and 90 miRNAs that target the 7 core CR‐DEGs. However, the specific regulatory mechanisms of TFs and miRNAs need to be further studied. We then predicted that 15 drugs could interact with IDH2, CDK1, and NDUFS1; thus, these drugs might be potential anti‐cuproptosis drugs.

Finally, we chose a testing dataset and clinical samples to validate the diagnostic value of the 7 core CR‐DEGs and immune infiltration in HBV‐ALF. Similar to the training dataset, we found that 6 of the 7 core CR‐DEGs, but not *DLAT*, presented the same changes in expression levels. Additionally, these CR‐DEGs also demonstrated excellent diagnostic value. In addition, the immune infiltration patterns of the test dataset were similar to those of the training dataset. In addition, the expression patterns of the samples from HBV‐ALF patients were similar to those of the GSE38941/GSE96851 samples. Therefore, we confirmed that the 7 core CR‐DEGs had remarkable diagnostic value and that HBV‐ALF had a unique immune response pattern.

To our knowledge, this is the first study to explore the immune response and cuproptosis in HBV‐ALF. Although some encouraging results were demonstrated, the limitations should not be ignored. First, the analysis might not be completely precise due to selection bias. A prospective study with a larger sample should be conducted to verify our results. In addition, additional mechanistic experiments, including studies in animals and humans, should be performed to elucidate the changes in CR‐DEGs and immune cells. In addition, the normal liver tissues in GSE96851 were from liver angioma, and whether angioma has any effect on the expression of CRGs is currently unknown.

## 5 Conclusion

In summary, we conducted a systematic bioinformatic analysis of HBV‐ALF and revealed that 7 core CR‐DEGs and infiltrating immune cells might play important roles in the physiological and pathological processes of HBV‐ALF. Seven core CR‐DEGs showed good diagnostic value in HBV‐ALF patients in our small cohort study. In addition, we found that some TFs, miRNAs, and drugs can target the core CR‐DEGs. These results provide a new perspective for the diagnosis of HBV‐ALF and novel potential therapeutic targets for cuproptosis in HBV‐ALF.

## Author Contributions

All authors contributed to the study's conception and design. Material preparation and data collection were performed by Jingwen Deng, Tao Zhu, and Yueyang Wang. Data analysis was performed by Jingwen Deng, Sitong Zhang, Hong Deng, and Huiqiang Cai. The first draft of the manuscript was written by Jingwen Deng, and reviewed by Guilan Quan, Tingting Peng, Chuanbin Wu, Huiqiang Cai, Chao Lu, and Xiaopeng Cai. The manuscript was supervised and finalized by Chao Lu and Xiaopeng Cai. All authors read and approved the final manuscript.

## Ethics Statement

Ethical approval for patients was obtained from the Ethics Committee of Sir Run Run Shaw Hospital, School of Medicine, Zhejiang University (Ethics Committee number: 2023‐926‐01). All participating patients were informed and the requirement for written informed consent was waived by the Ethics Committee due to the retrospective nature of the study. All procedures involving human subjects were conducted in accordance with the principles of the Declaration of Helsinki.

## Conflicts of Interest

The authors declare no conflicts of interests.

## Supporting information




**Supporting File**: exp270156‐sup‐0001‐SuppMat.docx.

## Data Availability

The datasets generated during and/or analysed during the current study are available from the corresponding author on reasonable request.
