# Supplementary material for: Cuproptosis‐Related Genes in Immune Infiltration and Diagnosis in Hepatitis B Virus‐Related Acute Liver Failure
Source: Exploration (Beijing). 2026 Mar 19;6(3):20240267. doi: 10.1002/EXP.20240267 (PMC13317792; doi:10.1002/EXP.20240267)
Supplement: Supplementary file 1 — Supporting File: exp270156‐sup‐0001‐SuppMat.docx. [file EXP2-6-20240267-s001.docx]

Supporting Information

**Cuproptosis-related genes in immune infiltration and diagnosis in hepatitis B virus-related acute liver failure**

**Table S1.** The detailed information of antibodies

| Names | Cat No. | Company |
| --- | --- | --- |
| 4 Hydroxynonenal antibody | Ab46545 | Abcam (Cambridge, MA, USA) |
| CD45 Polyclonal antibody | 20103-1-AP | ProteinTech Group (Chicago, IL, USA) |
| IDH2 Polyclonal antibody | 15932-1-AP | ProteinTech Group (Chicago, IL, USA) |
| MCM5 Polyclonal antibody | 11703-1-AP | ProteinTech Group (Chicago, IL, USA) |
| NDUFS1 Polyclonal antibody | 12444-1-AP | ProteinTech Group (Chicago, IL, USA) |
| Transketolase Polyclonal antibody | 11039-1-AP | ProteinTech Group (Chicago, IL, USA) |
| PDHX Polyclonal antibody | 10951-1-AP | ProteinTech Group (Chicago, IL, USA) |
| DLAT Polyclonal antibody | 13426-1-AP | ProteinTech Group (Chicago, IL, USA) |
| CDK1-Specific Polyclonal antibody | 19532-1-AP | ProteinTech Group (Chicago, IL, USA) |
| LIAS Polyclonal antibody | 11577-1-AP | ProteinTech Group (Chicago, IL, USA) |
| FDX1 Polyclonal antibody | 12592-1-AP | ProteinTech Group (Chicago, IL, USA) |

**Table S2.** The expression of 7 hub genes in other types of ALF

| Gene symbol | ischemia/reperfusion injury | | acetaminophen-induced liver injury | |
| --- | --- | --- | --- | --- |
|  | adj. P | Log2FC | adj. P | Log2FC |
| IDH2 | 0.698 | 0.1059623 | 0.0776 | -1.27 |
| MCM5 | 0.97 | 0.01307658 | 0.6478 | 0.345 |
| NDUFS1 | 0.11 | -0.2467143 | 0.1494 | -1.11 |
| TKT | 0.246 | -0.28217487 | 0.0951 | 1.1 |
| PDHX | 0.0779 | 0.66005617 | 0.2617 | -0.493 |
| DLAT | 0.492 | 0.10883366 | 0.988 | -0.0142 |
| CDK1 | 0.399 | 0.19228404 | 0.0983 | 1.58 |
